# Biological Activities and Pharmacokinetics of Praeruptorins from *Peucedanum* Species: A Systematic Review

**DOI:** 10.1155/2013/343808

**Published:** 2013-11-26

**Authors:** Parisa Sarkhail, Abbas Shafiee, Pantea Sarkheil

**Affiliations:** Pharmaceutical Sciences Research Center, Tehran University of Medical Sciences, 16th Azar Street, P.O. Box 14155-6451, Tehran 14176, Iran

## Abstract

Praeruptorins belonging to the angular-type pyranocoumarins are bioactive constituents that have been isolated from some *Peucedanum* species such as *P. praeruptorum*, which is used in traditional Chinese medicine for treatment of cold, cough, upper respiratory infections, and so forth. Many reports have demonstrated that the beneficial pharmacological effects of *P. praeruptorum* root on cardiovascular, pulmonary, immune, and nervous system diseases were attributed to the presence of praeruptorins. The aim of this review is to explain the recent efforts of scientists in pharmacological screening of natural and synthetic praeruptorin derivatives, studying the mechanisms of some praeruptorins action, pharmacokinetics, toxicity, and relevant structure-activity relationships. Based on reported data about the pharmacological properties of praeruptorins and semisynthetic derivatives of them, it is hopeful that in the near future more studies focus on the discovery of the new application and therapeutic uses of these bioactive compounds and understanding the specific mechanisms of them. The present discusses the reports on molecular and biological activities of praeruptorins of the genus *Peucedanum*, from 1976 onwards.

## 1. Introduction

Phytochemical investigations revealed that pyranocoumarins naturally exist in genus *Peucedanum* which includes more than 120 species and is widely distributed in Asia, Europe, and Africa. Praeruptorins with an angular-type, nonglycosidic pyranocoumarin (khellactone coumarins) structure were found in just a few *Peucedanum* species (see Table 1) such as *P. praeruptorum* Dunn., *Peucedanum japonicum* Thunb., and *P*. *formosanum* Hay. (Apiaceae) [1–8].

Khellactone (dihydroseselin) coumarins are notable because of their different activities, including calcium antagonist activity [9], antiplatelet aggregation [10], P-glycoprotein (Pgp) inhibitory ability [11], and anti-HIV effect [12]. For the first time, khellactone coumarins with 3′S, 4′S configuration (praeruptorins A, B, C, and D) are reported from dried roots of *P*. *praeruptorum* (Peucedani Radix). This species is known as a medicinal herb that is commonly used in traditional Chinese medicine (TCM) for treatment of cough and upper respiratory infections and as an antipyretic, antitussive, and mucolytic agent [13, 14]. In the folk medicine of Taiwan, Korea, Japan, and China two *Peucedanum* species, *P*. *japonicum* [15–17] and *P*. *formosanum* [4] are used for treatment of cough, cold, headaches, and so forth. The well-known traditional uses of these herbs are listed in Table 2. There have been many investigations about the pharmacological activities of *P*. *praeruptorum* and *P*. *japonicum* such as anticancer [11], antimicrobial [17], antidiabetic [18], antiobesity [19] and antioxidant [20] activities. In the Chinese Pharmacopeia, praeruptorin A and praeruptorin B are listed as the chemical markers for evaluating the quality of Peucedani Radix and related products [21]. Praeruptorins were found to be responsible for various pharmacological properties such as calcium antagonist activity [9], anti-inflammatory action [22], antiasthma, vasorelaxant and antiallergic effects [23, 24], cardiac protective [25, 26], hepatoprotective [27], antitumor [28–30], and antiplatelet aggregation activities [10].

This paper underlines some reported biological activities of praeruptorins and explains their possible mechanisms of action. In addition, it develops a better understanding of the relationship between structure and function of these bioactive components. Also, this review explains the pharmacokinetic-pharmacodynamic profile of praeruptorins in human and rat liver microsomes for next research directions in the further development of praeruptorins as potential therapeutic agents in clinical trial investigations. This review covers the literature available from 1975 to 12 April 2013. The information was collected from scientific journals via a library and electronic search (using Google Scholar, PubMed, Scopus, Web of Science, and Science Direct).

## 2. Chemical Structures of Praeruptorins

**Figure 1:**
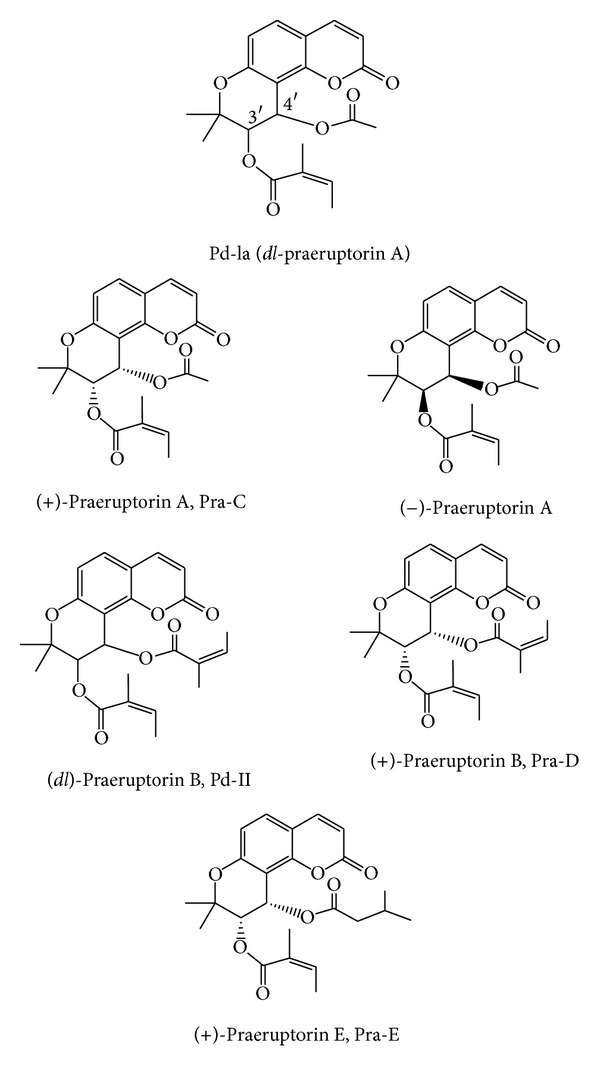
Molecular structures of praeruptorins from *Peucedanum* species.

Praeruptorins belonging to angular-type pyranocoumarins comprise a free sugar khellactone skeleton (dihydroseselin) with different substituents at the two stereogenic centers (C-3′ and C-4′). Based on their chemical structures, *cis*-khellactones are usually divided into two groups: (a) 3′*R*, 4′*R* or (b) 3′*S*, 4′*S* configuration. 3′*S*, 4′*S* configuration exists generally in *P*. *praeruptorum* and *P*. *japonicum* [30, 31]. (±)-Praeruptorin A or *dl*-praeruptorin A (Pd-la) with an angeloyl group on C-3′ and an acetyl group on C-4′ and (±)-praeruptorin B or *dl*-praeruptorin B (Pd-II, Anomalin) with angeloyl groups on C-3′ and C-4′, respectively, were found to be racemic to praeruptorin C (Pra-C, *d*Pra-A) and to praeruptorin D (Pra-D, *d*Pra-B). Study of chiral preference of praeruptorins indicated that dextrorotatory (+) isomers of praeruptorin A and praeruptorin B are naturally more abundant than their levorotatory (−) enantiomers in the Peucedani Radix [13]. Depending on the substituted groups on carbons 3′ and 4′, different khellactone compounds have been isolated from *Peucedanum* species (see Table 1). Chemical structures of praeruptorins are given in Figure 1.

### 2.1. Structure-Activity Relationships (SARs) of Praeruptorins

Recently, researchers have paid close attention to the synthesis of new derivatives of (3′*S*, 4′*S*)-*cis*-khellactone coumarins that show more potent activities against cancer cell lines and HIV [30]. For example, to find more Pgp modulators, many of Pd-la derivatives were synthesized. Subsequently, SARs studies of the derivatives confirmed that side chains at the C-3′ and C-4′ site seems a key role in keeping or enhancing the capacity of khellactone coumarin in modulating Pgp. First, aromatic acyls substitutions are more active than aliphatic acyls. Secondly, methoxylation on aromatic acyls remarkably increased the multidrug resistance (MDR) reversal activity, as shown in (±)-3′-O, 4′-O-dicinnamoyl-*cis*-khellactone (DCK), a semisynthetic potent MDR reversal agent [11].

**Figure 2:**
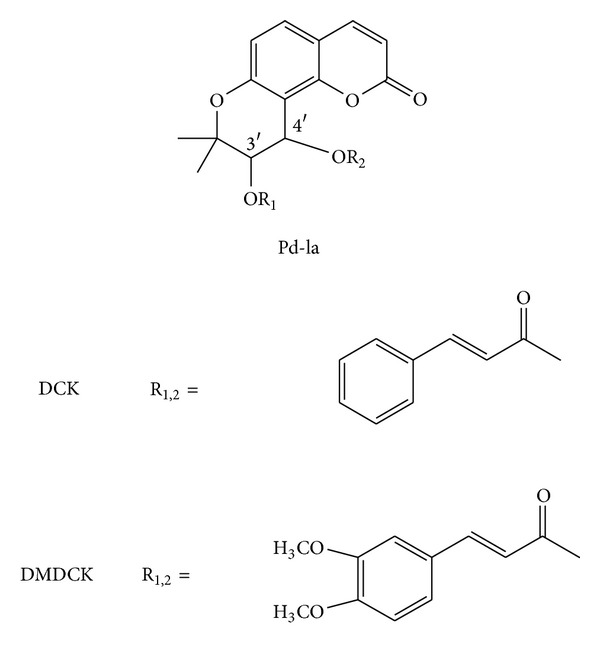
The molecular structures of semisynthetic praeruptorins from Pd-la. DCK: (±)-3′-O, 4′-O-dicinnamoyl-*cis*-khellactone; DMDCK: (±)-3′-O, 4′-O-*bis*(3, 4-dimethoxycinnamoyl)- *cis*-khellactone.

Replacement of aliphatic acyloxys by cinnamoyloxys at C-3′ and C-4′ in DCK could induce more hydrophobic property with a more planar configuration. Data showed that DCK was more effective than Pd-la and verapamil in reversing Pgp-MDR. Unlike Pd-la mechanism, DCK directly inhibited Pgp by a noncompetitive mechanism without inhibiting Pgp expression [31]. In a further study by Fong et al. [32], methoxy substitution of the aromatic rings significantly enhanced the interaction between Pgp and pyranocoumarins and influenced Pgp-MDR reversing action. (±)-3′-O, 4′-O-*bis*(3, 4-Dimethoxycinnamoyl)-*cis*-khellactone (DMDCK) was found as a good Pgp modulator because of higher Pgp-MDR reversing activity and relatively lower cytotoxicity. In Figure 2, the molecular structures of DCK and DMDCK are presented. Briefly, the role of aromatic acyloxy in the capacity of MDR-reversing of Pd-la derivatives follows the order 3, 4-dimethoxycinnamoyloxy and 3, 4-dimethoxybenzoyloxy > cinnamoyloxy and 4-methoxybenzeneacetyloxy > 4-methoxybenzoyloxy and 4-methoxycinamoyloxy > benzoyloxy and benzeneacetyloxy. Thirdly, the 3′, 4′*-cis*-configuration of aromatic acyls was more potent than their *trans*-isomers in the MDR-reversing ability of pyranocoumarins [11]. Also, many reported studies have explained that the rigid stereochemistry of 3′*R-* and 4′*R*-configured khellactone derivatives is essential for anti-HIV activity [33] and the presence of an acetoxy group in C3′ and C4′ of dihydroseselin structure was critical for relaxing smooth muscles [34].

## 3. Pharmacological and Toxicological Aspects

### 3.1. *Cardiopulmonary *and* Renovascular Protective* Effects of Praeruptorins

Vascular smooth muscle relaxation occurred through multiple mechanisms. Intracellular Ca^2+^ plays a critical role for the endothelium-independent relaxation of vascular smooth muscle so that blockage of either extracellular Ca^2+^ influx or internal Ca^2+^ release efficiently relaxes vascular smooth muscle. Hao et al. [35] have shown that Pd-la efficiently relaxed ileum and tracheal smooth muscles. (+)-Praeruptorin A (Pra-C) recovered the vascular hypertrophy by decreasing the area of smooth muscle cells (SMCs), collagen content, and Ca^2+^ in SMCs and by increasing production of nitric oxide (NO) in renovascular and spontaneously hypertensive rats. These findings showed that (+)-praeruptorin A (Pra-C) from Peucedani Radix has an important role in vascular smooth muscle relaxation. Both (+)-praeruptorin A and (−)-praeruptorin A produced a concentration-dependent relaxation in isolated rat aortic rings contracted by KCl. The action of (+)-praeruptorin A is more potent than (−)-praeruptorin A. The most important reason for this different activity is probably that (+)-praeruptorin A but not (−) praeruptorin A can well adjust to the pharmacophores of eNOS and activates NO/cGMP signaling pathway [36].

Zaho et al. [24] confirmed the relaxant effects of Pd-la and (+)-praeruptorin A in isolated rabbit tracheas and pulmonary arteries. Pd-la and (+)-praeruptorin A created significant relaxant effects in tracheal preparations constricted with 40 mM KCl or 10 **μ**M acetylcholine. The relaxation response to Pd-la or (+)-praeruptorin A in preparations constricted with KCl was more potent than that in preparations constricted with agonists like acetylcholine or phenylephrine in tracheas and pulmonary arteries. These pyranocoumarins at dose of 30 **μ**M absolutely relaxed tracheas constricted with 40 mM KCl. These results suggested that Pd-la and (+)-praeruptorin A are responsible for calcium antagonistic action and the presence an acetoxy group in C3′ and C4′ for dihydroseselin structure was critical for relaxing smooth muscles. Rao et al. [37] demonstrated that the effect of (+)-praeruptorin A and praeruptorin E in relaxing swine coronary artery and decreasing contractility in guinea pig left atria related to the calcium antagonist activity of these compounds, but their activity was less potent than nifedipine. (+)-Praeruptorin A at a dose of 2 mg/kg orally reduced the blood pressure in conscious normotensive, renal hypertensive rats and created significant drops in vertebral, left circumflex coronary and femoral vascular resistance in anesthetized dogs at doses of 20 and 100/**μ**g/kg *i.v*. A preliminary clinical trial confirmed that (+)-praeruptorin A is useful in the treatment of exertional angina pectoris, as a dosage of 100 mg daily of it could reduce chest pain, decrease the rate of periods of angina attacks, reduce the ST-segment changes, and finally decrease the dose of nitroglycerine consumption. In a further study, Kong et al. [38] indicated that some semisynthesized derivatives with C-3′, C-4′*trans*-configuration of (+)-praeruptorin A had a calcium antagonist activity, but they were not as potent as (+)-praeruptorin A [38]. (+)-Praeruptorin A isolated from *P*. *japonicum* root extract inhibited phenylephrine- (PE-) induced vasoconstriction in precontracted aortic rings in the concentration range of 10^−6^  −10^−4^ M. This activity was partially endothelium dependent and mediated by nitric oxide and cyclic GMP pathway. Indomethacin, a cyclooxygenase inhibitor, had no effects on the action of (+)-praeruptorin A. (+)-Praeruptorin A suppressed the high K^+^ (80 mM) induced and Ca^2+^-dependent contractions in a dose-dependent manner. On the basis of these results, (+)-praeruptorin A was a voltage-operated Ca^2+^ channel blocker rather than a receptor-operated Ca^2+^ channel blocker, although it weakly relaxed PE precontracted aortic rings in the presence of nifedipine. On the other hand, TEA (tetraethylammonium), a nonspecific K^+^ channel blocker, did not affect the vasodilatory activity of (+)-praeruptorin A against PE-induced contraction. Endothelium dependence and Ca^2+^ channel blockade are two mechanisms that are involved in the vasorelaxant effect of this compound [39]. In addition, Chang et al. [40] displayed that Pd-la is a calcium channel blocker and vasodilator in cardiohemodynamic modulation. Pd-la can probably control and regulate the expression of some immediate-early genes including IL-6, Fas, Bax, and Bcl-2 and reduce neutrophils infiltration. These mechaisms of action of Pd-la have the beneficial effects in ischemia-reperfusion myocardium and a significantly lower the incidence of cardiomyocyte apoptosis.

In another study, Pd-la showed cardiohemodynamic effects because of its Ca^2+^ channel blocker activity. The effects of Pd-la on mean aortic pressure and rate pressure product were about one-tenth as strong as those of diltiazem [41]. Pd-la showed a dose-dependent Ca^2+^ channel blocking effect in the single ventricular cells of guinea pig. In the presence of Pd-la (1, 10, and 100 **µ**M), inhibitory rates were 21%, 33.5%, and 45%, respectively [42–44]. In further investigation, Pd-la at a dose of 1.0 **µ**M with 30 min preventive perfusion decreased NF-*κ*B (nuclear factor kappa-light-chain-enhancer of activated B cells) activity from 0.98 ± 0.13 to 0.65 ± 0.17 (*P* < 0.05 versus solvent) and reduced tumor necrosis factor-*α* (TNF-*α*) from 13.7 ± 6.1 **µ**g/L to 9.4 ± 2.7 **µ**g/L (*P* < 0.01 versus solvent) expression in ischemia-reperfusion (I/R) myocardium, which might be one of the molecular mechanisms of Pd-la in cardioprotection [26]. On ischemic myocardial dysfunction in anesthetized dogs, injection of Pd-la (0.1–3.0 mg/kg *i.v.*) considerably and dose dependently improved coronary blood flow, reduced mean aortic pressure, and systemic vascular resistance with a minor rise in heart rate[9]. Furthermore, myocardial function was improved on regional myocardial dysfunction in anesthetized open-chest dogs by infusion of 0.15 mg/kg/min Pd-la for 30 minutes [24].

### 3.2. Anti-Inflammatory Effects of Praeruptorins

For the first time, Zhang et al. [45] described the significant antitussive and anti-inflammatory effects of the major constituent of Peucedani Radix collected from China. Yu et al. [46] explained that the anti-inflammatory effect of Pd-la in lipopolysaccharide- (LP-) stimulated RAW264.7 murine macrophage cell was related to inhibition of NF-*κ*B signal pathway activation. Pd-la inhibited NO, TNF-*α*, and interleukin-1 beta (IL-1b) production in a dose-dependent manner. Treatment with Pd-la at 25 mg/mL inhibited LPS-stimulated NO production up to 54%. Moreover, Pd-la treatment at the same dose caused 42% and 54% inhibition of TNF-*α* and IL-1b production, respectively. Pd-la also noticeably inhibited iNOS (inducible nitric oxide synthase) expression at the levels of protein and gene. Treatment with Pd-la showed inhibitory effects on the mRNA expression of TNF-*α* and IL-1b in LPS-stimulated RAW264.7 cells. They suggested that Pd-la may decrease the proinflammatory mediator release and exhibit anti-inflammatory effect.

Airway hyperreactivity is one of the remarkable characteristics of allergic asthma, leading to periodic bronchoconstriction and obstruction of symptoms [47]. The effect of coumarin-rich fraction from *P*. *praeruptorum* Dunn. (CPPD) on the lung resistance induced by acetylcholine chloride was used to evaluate the development of airway hyperreactivity in experimental mice. CPPD significantly suppressed airway hyperreactivity in a dose-dependent manner, and the effect of its high dosage was comparable with that of dexamethasone, which was consistent with previous reports of the attenuation of acetylcholine induced bronchoconstriction of rabbit by CPPD *in vitro* [24].

In 2012, Xiong and coworkers [48] confirmed the inhibitory effect of CPPD, containing praeruptorins, on allergic airway inflammation and T helper cell type two predominant responses in a mouse model. Pd-la has shown potent anti-inflammatory effect in a murine model of chronic asthma. Pd-la could regulate the NF(nuclear factor)-*κ*B pathway, and significantly reduced airway inflammation and airway hyperresponsiveness by reducing levels of some inflammatory mediators such as interleukin (IL)-4, IL-5, IL-13, leukotriene C4 (LTC4), eotaxin, and interferon gamma (INF-*γ*) in bronchoalveolar lavage fluid (BALF) and immunoglobulin (Ig) E in serum. Moreover, this compound suppressed expression of TGF (transforming growth factor) -*β*1, proteins Smad2/3, and upregulated the expression of Smad7 in lung tissue [49, 50]. Three praeruptorins C, D, and E were isolated from Peucedani Radix and exhibited anti-inflammatory activity in LPS-stimulated RAW264.7 murine macrophage cells through the inhibition of NF-*κ*B and STAT3 (signal transducer and activator of transcription 3) activation. All compounds significantly inhibited LPS-induced production of nitric oxide, IL-6, TNF-*α*, mRNA, and protein expressions of inducible nitric oxide synthase. Both Pra-D and Pra-E showed higher anti-inflammatory activities than Pra-C [22]. In a recent study, they examined the effects of praeruptorins Pd-la, C, D, or E, on LPS-induced pulmonary inflammation. Pretreatment with 80 mg/kg/orally of both Pra-D and Pra-E showed a significant lowering in the total cell and polymorphonuclear leukocytes (PMNs) counts, with decreasing the level of TNF-*α* and IL-6 in bronchoalveolar lavage fluid, while the effective doses of Pd-la and Pra-C were 320 mg/kg. Doses of 80 mg/kg Pra-D and Pra-E respectively, caused 51% and 56% decrease in TNF-*α* level and inhibited the release of IL-6 by 51% and 59%, respectively. In addition, Pra-D and Pra-E improved pathological changes in the lung and were able to suppress the NF-*κ*B activation acute lung injury induced by LPS and HCl [51].

### 3.3. Neuroprotective Effects of Praeruptorins

Pd-la from chloroform extract of the root of *P*. *japonicum* showed inhibitory activities on monoamine oxidase in mouse brain with IC_50_ value of 27.4 **μ**M [52]. Glutamate is the main excitatory neurotransmitter in the mammalian central nervous system (CNS) that excessive glutamate accumulation induces potent excitotoxicity in CNS and neuronal death both *in vitro* and *in vivo*. The N-methyl-D-aspartate of glutamate receptors (NMDARs) consists of a GluN2A subunit that promotes neuron protection, whereas GluN2B containing NMDARs mediates excitotoxicity [53]. Overstimulation of NMDARs causes overload of intracellular Ca^2+^ and then neuronal cell apoptosis [54]. In the latest study which was performed by Yang et al. [55], Pra-C at dose of 10 **μ**M showed a protective effect (92.5 ± 7.5%, *P* < 0.05 versus NMDA alone) against loss of cellular viability in excitatory neurotoxicity mediated by NMDA in primary cortical neurons. Pra-C increased the ratio of Bcl-2/Bax in NMDA-injured neurons and significantly inhibited neuronal apoptosis by reversing intracellular Ca^2+^ overload. Pra-C downregulated GluN2B-containing NMDA receptors by exposure to NMDA but did not affect the expression of GluN2A-containing NMDA receptors. These findings suggest a neuroprotective effect of Pra-C partially related to inhibiting the expression of GluN2B-containing NMDA receptors and regulating the Bcl-2 family. The effect of Pd-la on ATP sensitive potassium channels (K_ATP_ channel) in human cortical neurons was investigated by Zhang et al. [56]. K_ATP_ channels are widely present in CNS and the activation of KATP channel is one of the endogenous mechanisms of protection against ischemia or hypoxia. This study showed that Pd-la was a potassium channel opener (KCO) that increased the extracellular K^+^ level and caused cellular membrane hyperpolarization.

### 3.4. Cytotoxicity Effects of Praeruptorins

The median lethal dose (LD_50_) of the extraction of Peucedani Radix, which is mainly constituted by praeruptorins, has been reported over 5 g/kg in an acute toxicity testing in mouse [49, 50]. No behavioral effects or acute toxicity was observed after oral administration of various fractions: Pd-la and Pd-II from Peucedani Radix in mice. Also, delayed mortality was observed with EtOAc fraction and Pd-la only after intraperitoneal administration of high dose (1 g/kg). In the cytotoxicity *Artemia salina* test, the EtOAc fraction, Pd-la, and Pd-II showed 40.2, 121.2, and 34.5 **μ**g/mL LD_50_ values, respectively [57]. In 1990, Nishino and coworkers [58] investigated the effect of Pd-II *in vivo* tumor-promoting action of 12-O-tetradecanoylphorbol-13-acetate (TPA) initiated mouse skin. Pretreatment with a dose of 10 mumol/painting of Pd-II before the TPA treatment absolutely suppressed tumor formation up to 20 weeks of tumor promotion, without any toxicity.

The CHCl_3_ extract of the *P. japonicum* roots exhibited cytotoxic activity against the P-388 lymphocytic leukemia system with an ED_50_ 7.6 pg/mL. Moreover, Pra-C has shown cytotoxicity effect on the P-388 lymphocytic leukemia system in cell cultures with ED_50_ 2.6 pg/mL [59, 60]. The general pharmacological study of praeruptorins showed that oral administration of Pd-la and Pd-II did not provoke behavioral effects in mice; also no acute toxicity or mortality occurred at dose of 1 g/kg [57].

The cytotoxic activity of Pd-la was tested both on RAW264.7 cells and starch-elicited primary mouse peritoneal macrophages. Treatment of RAW264.7 with Pd-la did not influence the cell viability in dose range of 1–100 mg/mL. In addition, in primary mouse peritoneal macrophages, Pd-la did not show cytotoxic effect in dose range of 1–60 mg/mL. These results show the good safety profile of Pd-la *in vitro*, but *in vivo *safety profile needs to be further evaluated [38]. In the sulforhodamine B cytotoxicity assay, IC_50_ values of Pd-la on drug-sensitive KB-3-1 (human oral epidermoid carcinoma cell line) and drug-sensitive (MDR) KB-V1 (its multidrug resistant subline) cells were 41.91 ± 2.80 **μ**M and 17.26 ± 8.24 **μ**M, respectively. In comparison, the IC_50_ of doxorubicin was 0.06 ± 0.01 **μ**M for KB-3-1 and 3.05 ± 0.28 **μ**M for KB-V1. DNA fragmentation analysis confirmed that the Pd-la induced cell death via apoptotic mechanisms [61]. Recently, several natural and synthesized compounds have been screened for their Pgp-inhibiting activity. Although Pgp has an important role in protecting living tissues from damage by extruding xenotoxics out of cells, overexpression of this in tumor cells decreases the cellular concentration of anticancer drugs and leads to MDR [62]. Wu et al. [61] demonstrated that combining Pd-la with antitumor drugs such as doxorubicin (DOX), paclitaxel, puromycin, or vincristine in MDR KB-V1 cell line exhibited a synergistic effect, but not in drug-sensitive KB-3-1 cells. After 6 hours of Pd-la treatment, doxorubicin accumulation improved in KB-V1 cells by 25%. However, different doses of Pd-la after 24-hour incubation in KB-V1 cells could suppress the overexpression of Pgp in both protein and mRNA levels. Moreover, Pd-la rapidly reduced the cellular ATP contents in a dose-dependent manner in KB-V1 cells. Pd-la was shown to resensitize Pgp-overexpressing MDR (Pgp-MDR) cells by transiently depleting ATP and/or suppressing Pgp expression. Zhang et al. [63] suggested that Pd-la induced HL-60 (human promyelocytic leukemia) cell differentiation along both the myelocytic and monocytic lineages and both enantiomers are potential compounds for leukemia treatment. In this study, Pd-la inhibited proliferation in HL-60 cells in a time- and dose-dependent manner, a single dose of (20 **μ**/mL) Pd-la after 72 hours reduced cell growth by 90%, and cell cycle analysis increased in the part of G1 phase cells. Proliferation, differentiation, and apoptosis of cells are controlled by the interplay of three major mitogen-activated protein kinase (MAPK) pathways, namely, the extracellular signal regulated kinase (ERK1/2), c-Jun n-terminal kinase (JNK1/2), and p38 MAPK pathways. Another study revealed that Pra-C triggered mitochondria-mediated apoptosis in HL-60 cells and exhibited a time- and dose-dependent apoptotic effect in dose range of 10–30 **μ**g/mL on DNA fragmentation with involvement of ERK and JNK signal pathways in the process. Pra-C increased Box protein level and mitochondrial-bound Bax and finally elevated the Bax to Bcl-2 ratio which caused the loss of mitochondrial membrane potential and cytochrome *c* release [64].

Methanol extract of Peucedani Radix at 300 **µ**g/mL reduced growth of SGC7901 human gastric cancer cells by 51.2% (*P* < 0.01) due to the high concentration of Pd-la and Pd-II. Both Pd-la and Pd-II showed similar antiproliferative and cytotoxic activities on the SGC7901 cells after 24 h treatment in a dose-dependent manner, so that at concentration 100 **µ**M inhibited SGC7901 cell growth by 33.7% (*P* < 0.05). Additionally, Pd-la and Pd-II displayed cytotoxicity effect on SGC7901 cells by increasing lactate dehydrogenase (LDH) activities in a concentration-dependent manner in the cell culture supernatant after 24 hours of incubation with them. The maximum LDH-release was 29.7% for Pd-la (*P* < 0.01) and 26.5% for Pd-II (*P* < 0.01) in comparison with the control. The combination of the cytostatic drug DOX at 0.25 and 0.5 **µ**M and Pd-la at 100 **µ**M inhibited SGC7901 cell growth by 55.4% and 62.8% (*P* < 0.01 versus DOX alone) and, therefore, reduced the dose of DOX for decreasing severe side effects of chemotherapy [29].

### 3.5. Antimicrobial Activity of Praeruptorins

The EtOAc fraction and Pd-la from *P. praeruptorum* root had antimicrobial activity on *Streptococcus agalactiae*, with MIC (minimum inhibitory concentration) values of 250 and 100 **μ**g/mL, respectively [57].

### 3.6. Platelet Aggregation Inhibitory Activity of Praeruptorins

Pd-la and Pd-II showed an antagonistic activity specifically on platelet aggregation induced by platelet activating factor (PAF) [10].

## 4. Pharmacokinetics and Tissue Distribution Studies 

Ideally, drug metabolism and drug-drug interactions should be studied before the registration of bioactive agent. As hepatic cytochrome P450 (CYP) 3A4 enzyme has a key role in phase I xenobiotic metabolism, drug with CYP enzyme induction can result in faster drug metabolism and subtherapeutic drug concentrations, while drug enzyme inhibition can show drug accumulation and drug toxicity [65].

Coumarins are metabolized by a number of pathways such as 3-hydroxylation, 7-hydroxylation, and 3, 4-epoxidation [66]. Many studies have confirmed that 3-hydroxylation and 3, 4 epoxidation are the primary pathways of coumarins metabolism in rat liver microsomes and significantly catalyzed by CYP3A4 [67]. So far, some pharmacokinetic properties of *P. praeruptorum* and praeruptorins have just been described. However, their contribution to the *Peucedanum* species activities and *in vivo* active forms remains unclear [27]. As angular-type pyranocoumarins, Pd-la and Pd-II, are the main chemical markers used for quality control of *P. praeruptorum* herb and related products, pharmacokinetic properties of this plant are closely related to the absolute configurations of this type of coumarins that resulted from the configurations of two chiral carbon centers (C-3′ and C-4′) of metabolites (see Figure 1).

Several systematic pharmacokinetic studies were designed in rat and human liver microsomes to investigate the metabolic profiling of the main angular-type pyranocoumarins of *P*. *praeruptorum* and the relationship between these metabolites and efficiency of this herb. A rapid and sensitive analytical system such as high-performance liquid chromatography/ion trap tandem mass spectrometry (HPLC/IT-MS/MS) was applied to identify both large and small molecules from complex biological systems [27]. On the base of reported studies, *P*. *praeruptorum* root extract is metabolized mainly via cytochrome P450 isozymes (CYP) 3A1 and 3A2 in rats. Zhang et al. [68] measured the levels of Pd-la in plasma, urine, bile, tissues, and feces through a fast and sensitive liquid chromatography-tandem mass spectrometry (LC-MS/MS) method. The results confirmed that Pd-la was distributed and then eliminated quickly from rat plasma and indicated an apparent linear dynamics when were used in a range of 5–20 mg/kg. Tissue distribution study showed that Pd-la is principally distributed in blood-supply tissues such as heart, spleen, and lung with AUC (area under the curve) of 189%, 205%, and 134% of that in plasma, respectively, after intravenous (*i.v.*) administration. These findings confirmed that cardiovascular and respiratory systems are the main target organs of Pd-la. Also, low polarity of Pd-la permits it to cross the blood-brain barrier and show a noticeable level in the brain. Because of first hepatic metabolism pathway, Pd-la in the liver decreased faster than that in kidney and for this reason the total recoveries of Pd-la were low (0.097% in bile, 0.120% in urine, and 0.009% in feces) during 24 hours. Another study was performed on liver cirrhosis rats; they confirmed that the decreased metabolic clearance of Pd-la is at least partly due to the diminished levels of CYP3A1 and 3A2 [69]. The effect of praeruptorins Pd-la, C, D, and E on the activity of CYP3A4 mRNA expression, protein expression, and catalytic action through constitutive androstane receptor (CAR) mediated pathway in human colon adenocarcinoma cells (LS174T) was evaluated by Huang et al. [70]. The nuclear hormone receptor CAR has a play role in induction of drug metabolism and transport through regulation of the transcriptional activity of CYP3A4 gene *in vitro* and *in vivo*. Except Pra-E, praeruptorins significantly stimulated CAR and CYP3A4 receptor gene expression in a dose-dependent manner and therefore accelerated metabolism of CYP3A4 substrates and decreased adverse herb-drug interactions via increasing detoxification.

**Figure 3:**
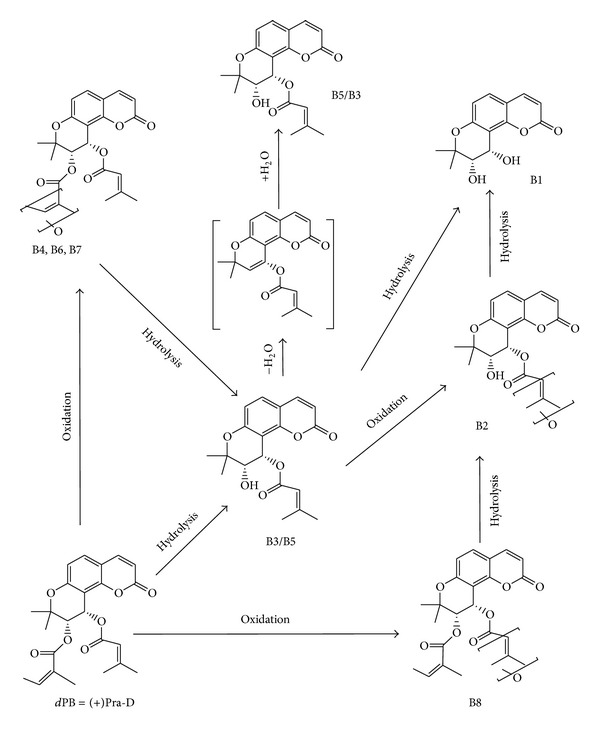
Proposed metabolic pathways of *d*PB (Pra-D) in liver microsomes of rats and humans. The intermediate is shown in brackets [72].

**Figure 4:**
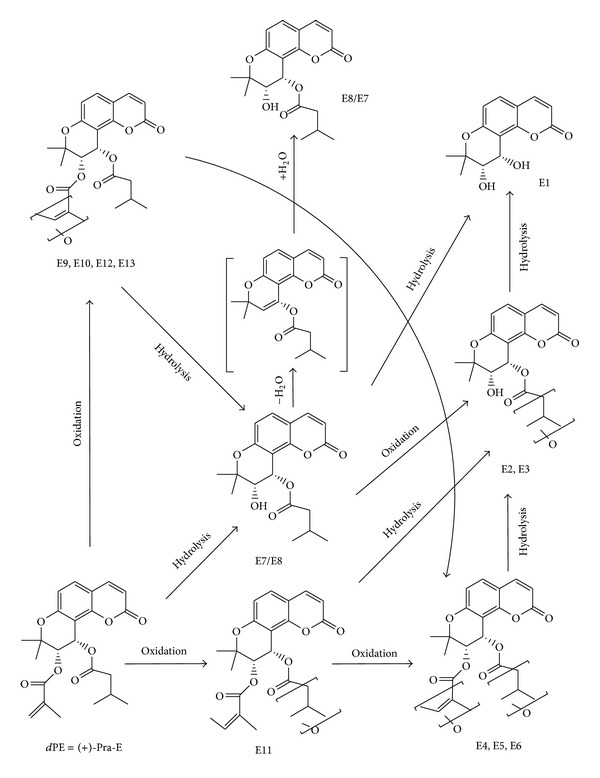
Proposed metabolic pathways of (+)-Pra-E in liver microsomes of rats and humans. The intermediate is shown in brackets [72].

Jing and coworkers [71] studied the transport and metabolism of Pd-la in human intestinal Caco-2 (heterogeneous epithelial colorectal adenocarcinoma) cells. Data showed that Pd-la was rapidly transported across Caco-2 cells and partly hydrolyzed and created two stereoisomers via removal of the acetyl group from C-4′ position. For the first time in 2011, Song et al. [72] investigated the metabolic profiles of Pra-D and (+)-Pra-E, two main bioactive constituents of Peucedani Radix, in rat and human liver microsomes (RLMs and HLMs). (+)-Pra-E was eliminated more rapidly than Pra-D in liver microsomes of both species. Similar biotransformation of (+)-Pra-E in liver microsomes resulted in 13 metabolites (E1–E13) that were similar in both RLMs and HLMs (see Figure 3). Moreover, metabolism of Pra-D was found to be very similar in both species. Therefore, the incubation of Pra-D produced eight similar metabolites (B1–B8) of Pra-D in RLMs and HLMs and metabolite B9 just detected in HLMs (see Figure 4). Tandem mass spectrometry on an MSD ion trap system (IT-MS/MS) joined with high-resolution mass measurement by time of flight mass spectrometry (TOF-MS) was applied for metabolite identification. In RLMs incubation, B3, one of the metabolites produced from hydrolysis of one angeloyl group from the C-3′ position of Pra-D, was the major metabolite, whereas B3 and B8 were found as two main metabolites in HLMs that were produced by monooxidation on the C-4 side chain of Pra-D. All the metabolites were generated in a NADPH-dependent manner. Two main metabolic pathways of Pra-D and (+)-Pra-E were shown to be oxidation and hydrolysis, and substituent at the C-3′ and C-4′ positions verifies the level and the type of reactions. Hydrolysis just started from the C-3′ substituent, but oxidation could be initiated at both substituents (C-3′ or C-4′) in metabolism pathway. In this study, they demonstrated that RLMs had more potential in catalyzing the metabolism of both Pra-D and (+)-Pra-E than HLMs and (+)-Pra-E was eliminated more rapidly than Pra-D in liver microsomes of both species.

Linag and coworkers [73] explained the pharmacokinetics and tissue distribution of Pra-D after administration of two doses of 10 and 20 mg/kg (*i.v.*) in rats. The plasma and tissues levels of Pra-D were evaluated by HPLC-UV method. The pharmacokinetic study showed that Pra-D is divided into two-compartment pharmacokinetic model including the fast distribution phase (*t*1/2*α*, 0.119–0.130 h) followed by a slow elimination phase (*t*1/2*β*, 2.408–2.640 h). One hour after Pra-D administration (20 mg/kg, *i.v.*), the maximum Pra-D level was found in all collected tissues. However, the maximum concentration of Pra-D was detected in the lung, followed by heart, liver, and kidney. Also, Pra-D was able to cross the blood-brain barrier and was detected in brain homogenate.

For the first time, Song et al. [27] determined stereoselectivity in Pd-la metabolism in RLMs and HLMs by combination of enzymatic hydrolysis and semipreparative chiral LC-MS/MS analysis of the metabolites. As angular-type pyranocoumarins have two stereogenic centers (C-3′ and C-4′), a chiral preference naturally caused the percent of levorotatory isomers of Pd-la and Pd-II to be less than their dextrorotatory enantiomers. These findings demonstrated that the hydrolysis, oxidation, and acyl migration were the principal pathways for (+) praeruptorin A (Pra-C) and (−)-praeruptorin A (Pd-la enantiomers). In RLMs, both enantiomers were eliminated more rapidly than in HLMs and both enantiomers exhibited stereoselective metabolism in RLMs and HLMs.

Basic hydrolysis of (+)-praeruptorin A produced (−)-*cis*-khellactone form, while (3′R, 4′R)-4′-angeloyl-khellactone and (3′R, 4′R)-3′-angeloyl-khellactone resulted from incubation of (−)-praeruptorin A in the rat plasma. These compounds were clearly identified by LC-MS/MS and NMR methods. The behavior of both enantiomers was different in the absence or presence of NADPH-regenerating system. In the absence of an NADPH-regenerating system, (+)-praeruptorin A remained unbroken; however, (−)-praeruptorin A yielded (3′R, 4′R)-4′-angeloyl-khellactone and (3′R, 4′R)-3′-angeloyl-khellactone by a carboxylesterase(s)-mediated process. On the other hand, in the presence of an NADPH-regenerating system, (−)-praeruptorin A produced nine metabolites in both species, whereas (+)-praeruptorin A generated 12 and six metabolites in RLMs and HLMs, respectively. In further study, the absolute configurations of other angular-type pyranocoumarin compounds were determined using similar method analysis. Hydrolysis of (±)-praeruptorin A (Pd-la), (+)-praeruptorin A (Pra-C), (−)-praeruptorin A, (+)-praeruptorin B (Pra-D), and (+)-Pra-E in the presence of an NADPH-regenerating system (hepatic phase I isozymes) generated *cis*-khellactone with the absolute configurations in both liver microsomes species [74].

**Figure 5:**
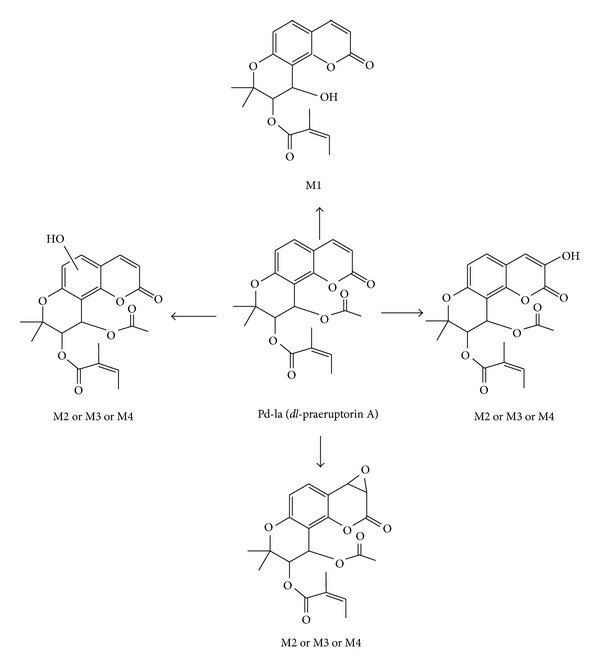
Proposed metabolic pathways of *dl*-praeruptorin A in rat liver microsomes.

**Figure 6:**
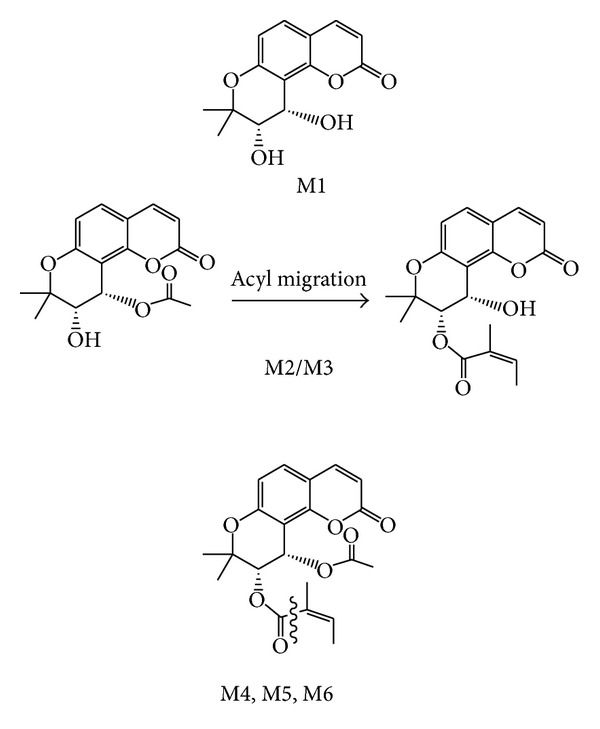
The metabolites of (+)-praeruptorin A produced via hydrolysis (M1–M3) and oxidation (M4–M6) in HLMs [76].

*In vitro* metabolism of Pd-la was investigated in rat liver microsomes by HPLC-ESI-MS to determine the CYP isoforms involved in the metabolism of Pd-la and detect and characterize the metabolites. The results showed that the metabolism of Pd-la in rat liver microsomes was dependent on the presence of NADPH and also the cytochrome P450 (CYP) and CYP3A1/2 involved in metabolism of it. The major metabolite of Pd-la was (M1) 3′-angeloyloxy-4′-hydroxyl-3′, 4′dihydroseselin (see Figure 5). The metabolites M2, M3, and M4 (hydroxyl-Pd-la), as minor metabolites, were unlike in the hydroxylation position in the Pd-la molecule. The polarity of Pd-la metabolites increased after hydroxylation and therefore the metabolites would be excreted easier than Pd-la from the body. The results confirmed that Pd-la metabolism is mediated by CYP3A1/2 and the metabolic changes in Pd-la may happen with other CYP3A inducers and/or inhibitors (drug-drug interactions) [75]. In a latest study, the kinetics of enzyme action and the main CYP450 isozyme(s) involved in the metabolism of (+)-praeruptorin A were considered in HLMs by a fast, sensitive, and reproducible UHPLC-QT-MS/MS (ultrahigh-performance liquid chromatography coupled with a hybrid quadruple-linear ion trap mass spectrometry) method. The metabolite of (+)-praeruptorin A is was formed *via* hydrolysis, oxidation, and hydrolysis followed by acyl migration (see Figure 6). Because (+)-praeruptorin A only catalyzed by CYP450 in HLMs, some disagreement results have been observed between previous studies and this work. (−)-*cis*-Khellactone as a major metabolite (M1) showed a biphasic kinetics in HLMs with high affinity (K_m1_ 0.02 **μ**M) and intrinsic clearance (CL_int1__,__*in* *vitro*_1.29 mL/min/mg protein), but other metabolites (M2–M6) followed typical Michaelis-Menten kinetics with minor affinity (K_m_ 3.85–39.13 **μ**M). Metabolism of (+)-praeruptorin A *via* CYP isozymes showed that recombinant human CYP3A4 had a maximum activity toward M1 and M4 formation, while it was CYP2C19 for M2/M3 and M5 and CYP2B6 for M6. However, CYP3A4 antibody inhibited all metabolites formation in a concentration range of 37–68%. These results confirmed an important role for CYP3A4 in human hepatic clearance of (+)-praeruptorin A; thus potential drug-drug interactions may occur when (+)-praeruptorin A is used with other CYP3A4 substrates [76]. Pharmacokinetic studies of praeruptorins are summarized in Table 3.

## 5. Conclusion

To date, most research studies conducted to find pharmacological and pharmacokinetic properties of praeruptorins have been focused on *P*. *praeruptorum*. This species is one of the traditional medicinal herbs in China which contains many active constituent coumarins like praeruptorins, and it has shown extensive pharmacological effects [13, 14]. Some of praeruptorins appear to be responsible for most of the activities of Peucedani Radix such as cardiovascular protection, antiasthma, and anticancer effects. Praeruptorins have been investigated for many years and numerous publications related to therapeutic activities of them were also reported [22–30]. As previously mentioned, there are several suggestions for the mechanism of cardioprotective, neuroprotective, anti-inflammatory, and anticancer activity of praeruptorins. In recent years, there has been increasing interest in the potential health benefits of natural and semisynthetic praeruptorin derivatives in multidrug resistance in cancer cells [11]. Praeruptorins showed several pharmacological effects that are attributed to their different chemical structures and configuration [11, 34]. In this paper, we reviewed the pharmacokinetic profile of praeruptorins in human and rat liver microsomes and the relationship between structure and function of these bioactive components. Pharmacokinetic studies showed that oxidation, hydrolysis, and acyl migration of C-3′ and/or C-4′ positions in hepatic microsome are the main metabolic pathways in metabolism of praeruptorins [75, 76]. Moreover, this review explained the pharmacological effects of *P. praeruptorum* and *P. japonicum* and praeruptorins based on cell culture experiments and animal studies. Although, according to some ancient ethnomedical traditions, *P. praeruptorum* and *P. japonicum* were used for a long time for prevention and treatment of some diseases [12–15], clinical trials to establish dose response, efficacy, and safety of praeruptorins as pharmaceuticals remain unclear. Therefore, future therapeutic investigations should focus on these objects and involve each praeruptorin for understanding the mechanisms of action in both animal models and human trials. Also, further studies are now required to establish whether praeruptorins have a clinically relevant metabolic interaction with CYP enzyme inducers and/or inhibitors to avoid or reduce CYP-mediated drug interactions and to optimiz dosing and duration of therapy to prevent subtherapeutic effects or toxicity.

## Figures and Tables

**Table 1:** The isolated praeruptorins from different *Peucedanum* species.

Compounds/synonyms	*Peucedanum* species	References
(±)-Praeruptorin A: Pd-Ia, *dl*-PA	*praeruptorum* *japonicum* *harry-smithii* var. *subglabrum* *luxurians *	[19, 42, 60, 63] [18] [3] [5]

(+)-Praeruptorin A: *d*PA, Pra-C	*praeruptorum *	[22, 24, 37]

(±)-Praeruptorin B: Anomalin, Pd-II, (±) praeruptorin D	*praeruptorum* *delavayi* *japonicum* *harry-smithii* var. *subglabrum *	[10, 22, 73] [6] [15] [3]

(−)-Anomalin	*wulongense *	[7]

(+)-Praeruptorin B: (+)-Anomalin, praeruptorin D, Pra-D	*formosanum* *praeruptorum *	[4] [45]

Praeruptorin E (Pra-E): wulongensin A	*praeruptorum* *zenkeri *	[22, 37] [8]

**Table 2:** The traditional medicinal uses and common names of some* Peucedanum* species including praeruptorins.

*Peucedanum* species	Common names	Part of use	Uses recorded and references
*P. formosanum* Hay. *P. terebinthaceum* subsp. *formosanum* (Hayata) Kitag.	Qian-Hu (in Taiwan)	Root	Coughs, fever, headache, and excessive sputum caused by colds [4].

*P. japonicum* Thunb.	Shoku-Bohfuu, Botan-Bofu (in Korea, Japan, China, and Taiwan)	Root	Sore throat, coughs, colds, headaches, and as an antifebrile and anodyne [12, 15, 17].

*P. praeruptorum* Dunn.	Peucedani Radix, Bai-Hua, Qian-Hu, BQ (in China), and Jeon Ho (in Korea)	Root	Treatment of respiratory diseases, pulmonary hypertension, angina pectoris, chest pain, dyspnea and upper respiratory infections, nonproductive cough with thick sputum, and as antitussive and mucolytic agents [13, 14, 22, 24, 37, 62, 72, 73].

**Table 3:** Summary of reported pharmacokinetic and tissue distribution studies of praeruptorins.

Compounds	Pharmacokinetic and tissue distribution studies	References
Pd-la	*In vivo* (in rat): a single dose administration (i.v.) of 5, 10, and 20 mg/kg Pd-la showed that it was quickly distributed and then eliminated from plasma. The main distribution tissues of Pd-Ia were spleen, heart, and lung; Pd-Ia was enabled to cross the blood-brain barrier due to low polarity.	[68]

Pd-la	*In vivo* (in rat): a single dose administration (i.v.) of 5 mg/kg Pd-la to rats with liver cirrhosis showed that the decreased metabolic clearance of Pd-la was at least partly due to the diminished levels of CYP3A1 and 3A2.	[69]

Pd-la	*In vitro* (in RLMs): CYP3A1/2 was the main isoform mediating both hydrolysis and oxidation. The major metabolite of Pd-la was (M1) 3′-angeloyloxy-4′hydroxyl-3′,4′dihydroseselin.	[75]

Pd-la, Pra-C, Pra-D, and Pra-E	*In vitro* (in human colon adenocarcinoma cells, LS174T): except Pra-E, praeruptorins significantly stimulated CAR and CYP3A4 receptor gene expression in dose-dependent manner.	[70]

Pd-la	*In vitro* (in human colon adenocarcinoma cells, Caco-2): Pd-la was rapidly transported across Caco-2 cells and partly hydrolyzed and created two stereoisomers via removal of the acetyl group from C-4′ position.	[71]

Pra-D and (+)-Pra-E	*In vitro* (in RLMs and HLMs): all the metabolites were generated in an NADPH-dependent manner. Oxidation and hydrolysis were two main metabolic pathways of Pra-D and (+)-Pra-E. RLMs had more potential in catalyzing metabolism of both Pra-D and (+)-Pra-E than HLMs. Metabolites B1 and E1 were identified as (−)-*cis*-khellactone.	[72]

Pra-D	*In vitro *(in rat): a single dose administration (i.v.) of 10, and 20 mg/kg Pra-D showed that it is divided into two-compartment pharmacokinetic model including the fast distribution phase (*t*1/2*α*, 0.119–0.130 h) followed by a slow elimination phase (*t*1/2*β*, 2.408–2.640 h).	[73]

Pra-C and (−)-praeruptorin A	*In vitro *(in RLMs and HLMs): in the absence of NADPH-regenerating system, Pra-C remained unbroken; however, (−)-praeruptorin A yielded (3′R, 4′R)-4′-angeloyl-khellactone and (3′R, 4′R)-3′-angeloyl-khellactone by a carboxylesterase(s)-mediated process. In RLMs, both enantiomers were eliminated more rapidly than in HLMs.	[27]

Pd-la, Pra-C, (−)-Pra-A, Pra-D, and (+)-Pra-E	*In vitro* (in RLMs and HLMs): hydrolysis of these praeruptorins in the presence of the NADPH-regenerating system (hepatic phase I isozymes) produced *cis*-khellactone with the absolute configurations.	[74]

Pra-C	*In vitro* (in HLMs): CYP3A4 was the main isoform mediating both hydrolysis and oxidation. (−)-*cis*-Khellactone as a major metabolite (M1) showed biphasic kinetics in HLMs.	[76]
